# CDK4/6 inhibition presents as a therapeutic option for paediatric and adult germ cell tumours and induces cell cycle arrest and apoptosis via canonical and non-canonical mechanisms

**DOI:** 10.1038/s41416-020-0891-x

**Published:** 2020-05-18

**Authors:** Margaretha A. Skowron, Marieke Vermeulen, Anna Winkelhausen, Teresa K. Becker, Felix Bremmer, Patrick Petzsch, Stefan Schönberger, Gabriele Calaminus, Karl Köhrer, Peter Albers, Daniel Nettersheim

**Affiliations:** 1 Department of Urology, Urological Research Lab, Translational UroOncology, University Hospital Düsseldorf, Düsseldorf, Germany; 2 Institute of Pathology, University Medical Center Goettingen, Goettingen, Germany; 3 Genomics and Transcriptomics Lab, Heinrich-Heine University Düsseldorf, Düsseldorf, Germany; 4 Department of Pediatric Hematology and Oncology, University Hospital Bonn, Bonn, Germany; 5 Department of Urology, University Hospital Düsseldorf, Düsseldorf, Germany

**Keywords:** Germ cell tumours, Drug screening, Translational research, Checkpoints, Molecular medicine

## Abstract

**Background:**

Germ cell tumours (GCTs) are the most common solid malignancies in young men. Although high cure rates can be achieved, metastases, resistance to cisplatin-based therapy and late toxicities still represent a lethal threat, arguing for the need of new therapeutic options. In this study, we analysed the potential of cyclin-dependent kinase 4/6 (CDK4/6) inhibitors palbociclib and ribociclib (PaRi) as molecular drugs to treat cisplatin-resistant and -sensitive paediatric and adult GCTs.

**Methods:**

Ten GCT cell lines, including cisplatin-resistant subclones and non-malignant controls, were treated with PaRi and screened for changes in viability (triphenyl tetrazolium chloride (XTT) assay), apoptosis rates (flow cytometry, caspase assay), the cell cycle (flow cytometry), the transcriptome (RNA-sequencing, quantitative reverse transcriptase-polymerase chain reaction (qRT-PCR) and on protein level (western blot). Expression profiling was performed on paediatric and adult GCT tissues (expression microarrays, qRT-PCR, immunohistochemistry, ‘The Cancer Genome Atlas’ database).

**Results:**

We demonstrate that adult GCTs highly express *CDK4*, while paediatric GCTs strongly express *CDK6* instead. Thus, both GCT types are potentially treatable by PaRi. GCTs presented as highly sensitive towards PaRi, which caused a decrease in viability, cell cycle arrest and apoptosis. Although GCTs mainly arrested in the G1/G0 phase, some embryonal carcinoma cell lines were able to bypass the G1/S checkpoint and progressed to the G2/M phase. We found that upregulation of *CDK3* and downregulation of many mitosis regulation factors, like the *HAUS* genes, might be responsible for bypassing the G1/S checkpoint and termination of mitosis, respectively. We postulate that GCT cells do not tolerate these alterations in the cell cycle and eventually induce apoptosis.

**Conclusion:**

Our study highlights PaRi as therapeutic options for cisplatin-resistant and -sensitive paediatric and adult GCTs.

## Background

Testicular type II germ cell tumours (GCTs), which are sub-divided into seminomas and non-seminomas, arise from the precursor lesion germ cell neoplasia in situ (GCNIS), which itself is the result of a defective primordial germ cell development.^1–4^ Type II GCTs represent the most common tumour of young men of age 17–45 years and thus are of pubertal or post-pubertal type.^1,2,4^ Especially in Western countries, incidence rates are rising steadily.^5^ Additionally, GCTs can be found in newborns and infants, where these pre-pubertal tumours are termed type I GCTs and present mainly as teratomas or yolk-sac tumours.^1,2,4^ In contrast to type II GCTs, type I GCTs rather develop from a flawed or misrouted early primordial germ cell and not from a GCNIS precursor lesion.

In general, type II GCTs are treated by orchiectomy followed by chemo- or radiotherapy. Despite high cure rates exceeding 90% over all stages, ~10–15% of patients with advanced disease relapse despite adequate multimodal first-line and first-salvage treatments and are deemed treatment refractory.^6–8^ Such patients are usually incurable and face an awful prognosis with a life expectancy of a few months only.^6–8^

Cyclin-dependent kinases (CDK) have been defined as key drivers of cell cycle progression in several cancer entities. An arrest in the cell cycle leads to suppressed tumour growth as well as metastasis. Thus, targeting the cell cycle via selective pharmacological inhibition is a promising therapeutic option. Previous studies proved the CDK4/6 pathway as being active in solid tumours, such as liposarcoma, rhabdomyosarcoma, non-small-cell lung cancer, glioblastoma, oesophageal cancer, melanoma and breast cancer.^9^ The role of the most prominent CDK4/6 inhibitors palbociclib (Ibrance), ribociclib (Kisqali) and abemaciclib (Verzenio) in monotherapy and combined therapy is under evaluation in several phase 1, 2 or 3 studies. Palbociclib and ribociclib (PaRi) are reversible orally active pyridopyrimidine, which bind to the adenosine triphosphate binding site of the two CyclinD (CCND)-dependent kinases.^10^ Trials observed nearly indistinguishable clinical results between palbociclib and ribociclib.^11^ In 2017, the Food and Drug Administration approved PaRi in combination with aromatase inhibitors and abemaciclib plus fulvestrant as initial endocrine-based therapy for women with hormone receptor-positive, human epidermal growth factor receptor 2-negative advanced or metastatic breast cancer. These inhibitors eventually block the CCND–CDK4/6 complex, resulting in hypophosphorylation of the retinoblastoma (RB) protein.^12^ As a consequence, the transcription factor E2F cannot be cleaved from the RB complex and transcription of genes involved in cell cycle progression diminishes, resulting in a cell cycle arrest in the G1/G0 phase.^10,13^ In GCTs, an ‘unorthodox’ spectrum of defects within the CDK4/6-RB1 pathway has been described, that is, expression of RB1 on the messenger RNA (mRNA) level in all seminomatous and non-seminomatous GCTs, but detectable expression of RB1 on protein level only in teratomas, frequent deletions of RB1 and low expression of P16INK4A in GCNIS, seminomas and embryonal carcinomas (ECs) and overexpression of CyclinD2 (*CCND2*), as well as expression of CDK4 in GCNIS and invasive GCTs.^14–19^

In this study, we investigated the therapeutic potential of CDK4/6 inhibition in cisplatin-resistant and -sensitive paediatric and adult GCTs.

## Methods

### Cell culture

In this study, the following GCT cell lines were used: TCam-2 (seminoma), 2102EP, NCCIT, NT2/D1 (all EC), JAR, JEG3 and BeWo (all choriocarcinoma). In the cisplatin-resistant GCT sublines 2102EP-R, NCCIT-R and NT2/D1-R, cisplatin resistance was provoked by cultivating these EC cell lines for a prolonged time with increasing sub-lethal cisplatin concentrations as published.^20,21^ All GCT cell lines, MPAF fibroblasts and FS1 Sertoli cells were cultivated as described previously.^22,23^ The cell lines HaCat, HEK293 and VHF fibroblasts were cultivated in Dulbecco’s modified Eagle’s medium (10% foetal calf serum (FCS), 1% penicillin/streptomycin) and THP-1 cells in RPMI medium (10% FCS, 1% penicillin/streptomycin, 200 mM L-Glutamine) at 37 °C and 5% CO_2_. Short tandem repeat profiles of all cell lines are checked on a regular basis and are available upon request. For CDK4/6 inhibition, PaRi were dissolved in distilled water (pH 3) or dimethyl sulfoxide, respectively.

### Measurement of cell viability and Caspase-3/7 activity

The triphenyl tetrazolium chloride (XTT) assays were performed as described previously.^23^ In summary, 4000 cells were plated onto 96-well plates 24 h before PaRi or solvent application. In a total of 96 h, every day viability was screened by adding 50 μl XTT (1 mg/ml) plus 0.5 μl phenazine methosulfate (1.25 mM) (both from Sigma-Aldrich, Taufkirchen, Germany) and measuring absorbance 4 h later in a ultraviolet–visible spectrometer (450 vs. 650 nm, BMG Labtech, Ortenberg, Germany). Each time point/concentration was measured in quadruplicates. LD_50_ (lethal dose, 50%) doses were calculated using the GraphPad Prism software version 6. Relative Caspase-3/7 activity was adjusted to the relative cell viability as measured by Caspase-Glo-3/7 Assay and CellTiter-Glo Luminescent Cell Viability Assay (both Promega, Madison, WI, USA), respectively, and performed according to the manufacturer’s protocols.

### Flow cytometry (apoptotic and mitotic cells, cell cycle phase profiles)

Flow cytometry analyses of apoptosis rates and cell cycle distribution was performed by Annexin V/propidium iodide (PI) or PI staining, respectively. Briefly, for PI flow cytometry, cells were washed once with phosphate-buffered saline (PBS), fixed with Nicoletti buffer (0.1% Triton-X100, 0.1% sodium-citrate dehydrate dissolved in PBS), and stained with 50 μg/ml PI (Sigma-Aldrich) for 10 min at room temperature (RT). For apoptosis measurement, cells were washed once with Annexin V-binding buffer (10 mM HEPES pH 7.4, 150 mM sodium chloride, 5 mM potassium chloride, 5 mM magnesium chloride, 1.6 mM calcium chloride dissolved in H_2_O) before incubation with 5 μl Annexin V (ImmunoTools, Friesoythe, Germany) and 50 μg/ml PI (Sigma-Aldrich). For phospho-H3 (pH3) antibody staining, cells were permeabilised with 0.5% Triton X-100 before an 1 h incubation with a pH3 antibody at RT. Secondary antibodies and 50 μg/ml PI were added subsequently before flow cytometric measurement. For antibody details see Table 1. All flow cytometry analyses were performed using the MACSQuant Analyser (Miltenyi Biotech, Bergisch Gladbach, Germany). A total of 50,000 cells was measured for each sample/experiment.

**Table 1:** Antibodies used in this study.

Antibody	Company	Clone	Order no.	Dilution	Application
Primary antibodies					
β-ACTIN	Sigma-Aldrich	AC-15	A5441	1:20000	Western blot
GAPDH	Abcam	6C5	ab8245	1:30000	Western blot
RB1	Cell Signalling Technology	D20	9319S	1:500	Western blot
pRB1	Abcam	ab4784	40615	1:1000	Western blot
CDK4	Santa Cruz Biotechnology	DCS31	sc-56277	1:1500	Western blot
CDK4	Thermo Fisher Scientific	DCS31	AHZ0202	1:40	Immunohistochemistry
CDK6	Santa Cruz Biotechnology	B-10	sc-7961	1:1000	Western blot
CyclinD1	Cell Signalling Technology	92G2	2978	1:1000	Western blot
CyclinD2	BD Pharmingen	G132-43	554200	1:500	Western blot
Cleaved PARP	Cell Signalling Technology	D214	9541S	1:500	Western blot
PARP	Cell Signalling Technology	46D11	46D11	1:1000	Western blot
Aurora kinase B	BD Pharmingen	AIM-1	611083	1:500	Western blot
Ki-67	Santa Cruz Biotechnology	MIB-1	sc-101861	1:50	Immunofluorescence
Phospho-H3	Cell Signalling Technology	D7N8E	53348	1:1600	Flow cytometry
Secondary antibodies					
Polyclonal rabbit anti-mouse HRP	Dako		P026002-2	1:1000	Western blot/IHC
Polyclonal goat anti-rabbit HRP	Dako		P044801-2	1:2000	Western blot/IHC
Goat anti-mouse IgG (H+L) Alexa Fluor 488	Thermo Fisher Scientific		A11029	1:2000	Immunofluorescence
Goat anti-rabbit IgG (H+L) Alexa Fluor 488	Thermo Fisher Scientific		A11034	1:2000	Immunofluorescence

### RNA and protein isolation

RNA was isolated (including DNase I digestion) using the RNAeasy Mini Kit (Qiagen, Hilden, Germany) according to the manual.

Proteins were extracted by RIPA buffer containing 10% protease and phosphatase inhibitors (Sigma-Aldrich). Protein concentrations were assessed by the BCA Protein Assay Reagent Kit (Thermo Fisher Scientific, Dreieich, Germany).

### Western blot

Twenty micrograms of whole protein lysates were used for western blotting. Staining with Ponceau S solution (Sigma-Aldrich); 0.5% in 5% acetic acid) confirmed a correct membrane transfer. Membranes were blocked in 5% non-fat milk or 5% bovine serum albumin (BSA) in PBS + 1% Tween-20 (PBST) for 1 h and then incubated with primary antibodies overnight at 4 °C. Secondary horseradish peroxidase (HRP)-conjugated antibodies were incubated for 1 h at RT. The ChemiDoc Imaging System (Bio-Rad, Düsseldorf, Germany) was used for imaging. For antibody details see Table 1.

### Immunofluorescence staining

Cells were seeded in a 96-well plate and fixed with 4% formaldehyde (10 min) before permeabilisation with 0.2% Triton X-100 in PBS (5 min) with subsequent blocking in 1% BSA, 5% goat serum, 0.3 M glycine and 0.1% Tween-20 in PBS (1 h). Ki-67 antibody was incubated overnight at 4 °C before the addition of secondary antibody (1 h) and counterstaining with 0.5 μg 4′,6-diamidino-2-phenylindole (5 min). For antibody details see Table 1.

### Immunohistochemistry

Immunohistochemical reactions were performed on 4-μm formalin-fixed and paraffin-embedded tissue sections as published previously.^24^ Antigen retrieval was carried out at 98 °C in ethylenediaminetetraacetic acid buffer (pH 9; 20 min). Primary antibodies were incubated for 30 min at RT. Afterwards, sections were incubated with an HRP-labelled secondary antibody at RT for 25 min. The substrate ‘DAB + Chromogen’ system was applied to visualise the target antigen (Dako, Hamburg, Germany). For antibody details see Table 1.

### Quantitative reverse transcriptase-polymerase chain reaction

1 µg of RNA was in vitro transcribed into complementary DNA (cDNA) using 1 µl dNTP-Mix (10 mM), 1 µl Oligo(dT)18 Primer (0.5 µg/µl), 4 µl RT Buffer (5×), 1 µl Maxima H Minus Reverse Transcriptase (200 U/µl) and 0.5 µl RiboLock RNase Inhibitor (40 U/µl) (all Thermo Fisher Scientific) on a S1000 cycler (Bio-Rad) at 50 °C for 30 min. Further, quantitative reverse transcriptase-polymerase chain reaction (qRT-PCR) runs were performed on a 384-well C1000 cycler (both Bio-Rad). In general, all samples were analysed in technical triplicates using 7.34 ng of cDNA for each replicate and the SYBR-green-based Luna Universal qPCR Master Mix (New England Biolabs, Frankfurt a. M., Germany). At the end of each run, melting-curve analyses were performed. Oligonucleotide sequences are given in Table 2.

**Table 2:** Oligonucleotides used in this study.

Gene	Forward primer	Reverse primer	Temp.	Cycles
*APAF1*	ACAATGCTCTACTACATGAAGGATATAAAGA	CACTGGAAGAAGAGACAACAGGAA	60 °C	45
*AURKB*	CGCAGAGAGATCGAAATCCAG	AGATCCTCCTCCGGTCATAAAA	60 °C	45
*CCNA1*	TAGACACCGGCACACTCAAG	AGGAGAGATGAATCTACCAGCAT	60 °C	45
*CCNA2*	CGCTGGCGGTACTGAAGTC	GAGGAACGGTGACATGCTCAT	60 °C	45
*CCNB1*	AGCTGCTGCCTGGTGAAGAG	GCCATGTTGATCTTCGCCTTA	60 °C	45
*CCNB2*	CCGACGGTGTCCAGTGATTT	TGTTGTTTTGGTGGGTTGAACT	60 °C	45
*CCND1*	TACTACCGCCTCACACGCTTC	TTCGATCTGCTCCTGGCAG	60 °C	45
*CCND2*	GCTGGCTAAGATCACCAACACA	CCTCAATCTGCTCCTGGCAA	60 °C	45
*CCND3*	TACCCGCCATCCATGATCG	AGGCAGTCCACTTCAGTGC	60 °C	45
*CCNE1*	CCACACCTGACAAAGAAGATGATGAC	GAGCCTCTGGATGGTGCAATAAT	60 °C	45
*CDK1*	AAACTACAGGTCAAGTGGTAGCC	TCCTGCATAAGCACATCCTGA	60 °C	45
*CDK4*	ATGGCTACCTCTCGATATGAGC	CATTGGGGACTCTCACACTCT	60 °C	45
*CDK6*	CTGAATGCTCTTGCTCCTTT	AAAGTTTTGGTGGTCCTTGA	60 °C	45
*CDKN1A*	TGGAGACTCTCAGGGTCGAAA	GGCGTTTGGAGTGGTAGAAATC	60 °C	45
*DDIT4*	GGACCAAGTGTGTTTGTTGTTTG	CAC CCA CCC CTT CCT ACT CTT	60 °C	45
*E2F1*	ACGTGACGTGTCAGGACCT	GATCGGGCCTTGTTTGCTCTT	60 °C	45
*E2F3*	AAAGCCCCTCCAGAAACAAGA	CCTTGGGTACTTGCCAAATGT	60 °C	45
*E2F4*	CACCACCAAGTTCGTGTCCC	GCGTACAGCTAGGGTGTCA	60 °C	45
*E2F5*	TGGCAACTCAAAATCTGCCTG	TTGTAGTCATCTGCCGGGGTA	60 °C	45
*E2F6*	TCCATGAACAGATCGTCATTGC	TCCGTTGGTGCTCCTTATGTG	60 °C	45
*E2F7*	TAGCTCGCTATCCAAGTTATCCC	CAATGTCATAGATGCGTCTCCTT	60 °C	45
*FAS/APO-1*	AGCTTGGTCTAGAGTGAAAA	GAGGCAGAATCATGAGATAT	60 °C	45
*FOS*	GAGAGCTGGTAGTTAGTAGCATGTTGA	AATTCCAATAATGAACCCAATAGATTAGTTA	60 °C	45
*FOXM1*	CGTCGGCCACTGATTCTCAAA	GGCAGGGGATCTCTTAGGTTC	60 °C	45
*GAPDH*	TGCCAAATATGATGACATCAAGAA	GGAGTGGGTGTCGCTGTTG	60 °C	45
*RB1*	ATGGCTACCTCTCGATATGAGC	GCTTGGTTAACTTGGGAGAA	60 °C	45
*UHRF1*	GCCATACCCTCTTCGACTACG	GCCCCAATTCCGTCTCATCC	60 °C	45
*VEGFA*	AGGGCAGAATCATCACGAAGT	AGGGTCTCGATTGGATGGCA	60 °C	45

### Affymetrix/Illumina HT-12v4 expression arrays and Illumina 450k DNA methylation array

The Affymetrix expression array analysis of GCT tissues (GCNIS, *n* **=** 3; seminomas, *n* **=** 4; ECs, *n* **=** 3; teratomas, *n* **=** 3; normal testis tissue (NTT), *n* **=** 4) was performed previously and re-analysed in the context of this study.^25^ Illumina microarray expression data of parental GCT cell lines (TCam-2, *n* **=** 5; 2102EP, *n* **=** 5, NCCIT, *n* **=** 4; JAR, *n* **=** 2; FS1, *n* **=** 4; MPAF, *n* **=** 4) was extracted from previous studies available via Gene Expression Omnibus (GEO, ncbi.nlm.nih.gov/geo/) (GSE71239, GSE71269, GSE79065, GSE60698).^23,26–29^ The Illumina 450k DNA methylation array was generated in a previous study and re-analysed in the context of this study (GSE76709).^30^

### RNA-sequencing

RNA samples used for transcriptome analyses were quantified (Qubit RNA HS Assay, Thermo Fisher Scientific) and quality was measured by capillary electrophoresis using the Fragment Analyser and the ‘Total RNA Standard Sensitivity Assay’ (Agilent Technologies Inc., Santa Clara, USA). The library preparation was performed according to the manufacturer’s protocol (VAHTS Stranded mRNA-Seq Library Prep Kit). Three hundred nanograms of total RNA was used for mRNA capturing, fragmentation, synthesis of cDNA, adapter ligation and library amplification. Bead purified libraries were normalised and finally sequenced on the HiSeq 3000/4000 System (Illumina Inc., San Diego, USA) with a read setup of 1 × 150 base pairs (bp). The bcl2fastq tool was used to convert the bcl files to fastq files as well for adapter trimming and demultiplexing. Data analyses on fastq files were conducted with CLC Genomics Workbench (version 12.0.2, Qiagen). The reads of all probes were adapter (Illumina TruSeq) and quality trimmed (using the default parameters: bases below Q13 were trimmed from the end of the reads, ambiguous nucleotides max. 2). Mapping was done against the *Homo sapiens* (hg38) (25 May 2017) genome sequence. Statistical differential expression tests were determined using the ‘Differential Expression in Two Groups’ tool (version 1.02). The resulting *p* values were corrected for multiple testing by false discovery rate and Bonferroni correction. A *p* value of ≤0.05 was considered significant.

### Online analyses tools

Venn diagrams were generated using Venny 2.1 (https://bioinfogp.cnb.csic.es/tools/venny/).^31^ The STRING algorithm was used to predict interactive networks from RNA-seq data (https://string-db.org).^32^ Functional annotation analyses were performed by DAVID (https://david.ncifcrf.gov/home.jsp).^33,34^ In functional annotation analyses of commonly deregulated genes in GCT cells, only categories (UP_Keywords) with at least five members and *p* values ≤0.05 were considered significant. For functional annotation analysis of genes deregulated in each GCT cell line, only categories (UP_Keywords) with at least ten members and a *p* value ≤0.05 was considered significant. Only genes related to an official human gene symbol were included. ‘The Cancer Genome Atlas’ (TCGA) datasets were analysed for isoform/gene expression, DNA methylation and copy number alterations (CNAs) using the UCSC Xena browser (https://xena.ucsc.edu) and the cBioPortal (https://www.cbioportal.org).^35–37^

## Results

**Fig. 1:**
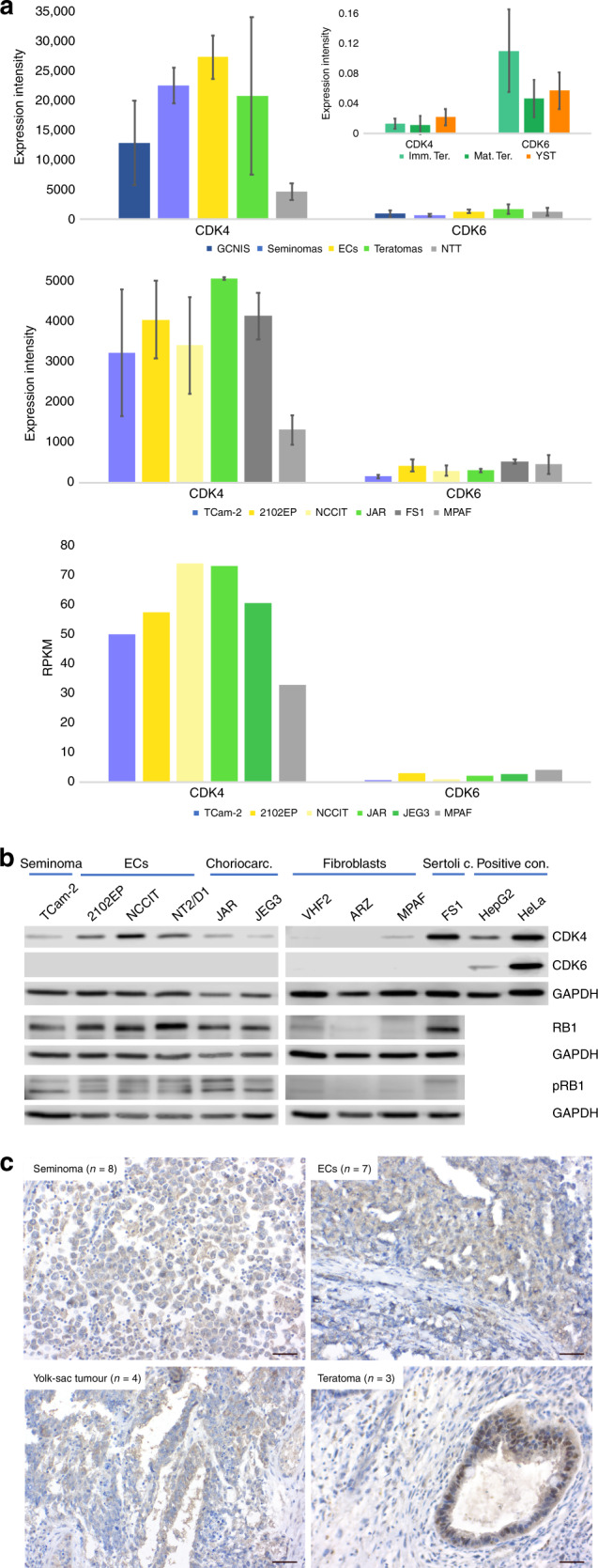
Expression of *CDK4/6* in GCT tissues and cell lines. **a** Analysis of *CDK4/6* expression in GCT tissues (type II GCTs, upper panel, Affymetrix microarray; type I GCTs, inlay in upper panel, qRT-PCR) and cell lines (middle panel: Illumina microarray; lower panel: RNA-seq data, RPKM = reads per kilobase million). As controls, normal testis tissue (NTT), the Sertoli cell line FS1 and fibroblasts (MPAF) were included. Standard deviation is given above bars. **b** Western blot analysis of CDK4, CDK6, RB1 and phospho-RB1 (pRB1) protein levels in GCT cell lines and controls (fibroblasts, Sertoli cells). HepG2 and HeLa cells served as positive controls for CDK4 and CDK6. GAPDH was used as housekeeper and for normalisation. **c** Immunohistochemical staining of CDK4 in GCT tissues (seminoma, EC, yolk-sac tumour and teratoma). Scale bar: 500 μm.

In this study, we analysed the potential of CDK4 and CDK6 inhibitors palbociclib (PF-00080665, Pfizer Ltd.) and ribociclib (GST0000015996, Novartis Pharma AG) as therapeutic options for cisplatin-resistant and -sensitive GCTs. First, by re-evaluating microarray data of GCT tissues and cell lines, as well as by western blot analyses of GCT cell lines, we screened for expression of *CDK4*/CDK4 and *CDK6*/CDK6 on RNA and protein level, respectively (Fig. 1a, b). *CDK4*/CDK4 was highly expressed in GCT tissues (GCNIS, seminomas, ECs, teratomas) and cell lines (TCam-2, seminoma; 2102EP, NCCIT, both EC; JAR, choriocarcinomas) (Fig. 1a, b). Additionally, *CDK4* expression was detectable in NTT, Sertoli cells (FS1) and fibroblasts (MPAF) (Fig. 1a). In contrast, *CDK6*/CDK6 expression was absent or very weak in all tissues and cell lines analysed (Fig. 1a, b). Interestingly, in paediatric type I GCTs, expression of *CDK6* was higher than *CDK4* (Fig. 1a, inlay in upper panel). We also confirmed CDK4 expression on protein level by immunohistochemistry of formalin-fixed-paraffin-embedded GCT tissues and found mainly cytoplasmatic, but also nuclear staining in seminomas (*n* = 8), ECs (*n* = 7), yolk-sac tumours (*n* = 4) and teratomas (*n* = 3) (Fig. 1c). Using the UCSC Xena browser, we screened the TCGA dataset of testicular GCT tissues for expression of *CDK4/6* and their different isoforms in GCT tissues.^36^ The isoforms *CDK4-201* (ENST00000257904.11) and *CDK6-201* (ENST00000265734.8) seemed to be the predominantly expressed isoforms in GCT (purple) and normal testis tissue (green) (Supplementary Fig. S1A, B). We stratified the TCGA dataset of 156 samples into a seminoma expression signature (*SOX17*, *PRAME*, *PRDM1* positive; *SOX2*, *DNMT3B*, *GAL* negative) and an EC expression signature (*SOX2*, *DNMT3B*, *GAL* positive; *SOX17*, *PRAME*, *PRDM1* negative) (Supplementary Fig. S1C). Additionally, we included *AFP* and *β-hCG* (*CGB*), demonstrating that *AFP* positivity is associated with the EC signature (indicative of yolk-sac tumour components), while *β-hCG* positivity can be found in both expression signatures (indicative of choriocarcinoma component in EC signature and choriocarcinoma/trophoblast component in seminoma signature) (Supplementary Fig. S1C). *CDK4* was strongly expressed in both seminoma and EC signatures, while *CDK6* expression was less intense compared to *CDK4*. Interestingly, in contrast to *CDK4*, *CDK6* positivity was clearly associated with a non-seminomatous signature (Supplementary Fig. S1C).

We asked, if DNA methylation might influence *CDK4*/*6* expression in GCTs (Supplementary Fig. S2A). In *CDK4*-positive and -negative GCT tissues from the TCGA cohort, we found mainly hypomethylated CpG dinucleotides around the transcription start site (TSS) and the gene body of *CDK4* (Supplementary Fig. S2A). In *CDK6*-positive and -negative GCT tissues, the TSS presented as hypomethylated, while we found a region ranging from the middle of the gene body to the 3′ end of the gene, where high DNA methylation levels correlated to high *CDK6* expression (and vice versa) (Supplementary Fig. S2A, black box). Up to now, the consequence of this finding remains elusive. These methylation dynamics/profiles could also be found in GCT cell lines (Supplementary Fig. S2B). In conclusion, although the *CDK4* gene locus seems rather hypomethylated, while the *CDK6* gene body seems hypermethylated, no correlation between *CDK4/6* expression and DNA methylation became obvious. At the most, one might speculate that the high *CDK6* gene body methylation might limit expression of *CDK6* and might be responsible for the low *CDK6* expression in GCTs compared to *CDK4* expression.

In GCT cell lines, we also screened for the expression of the CDK4 targets RB1 and phospho-RB1 (pRB1) (Fig. 1b). On protein level, RB1 and pRB1 were highly detectable in GCT cell lines, while the control cells showed only moderate RB1 and weak pRB1 levels (Fig. 1b).

We screened three studies of GCT tissues for common CNAs (amplifications and deletions) in seminomas and non-seminomas (TCGA Pan-Cancer Atlas, Firehose Legacy^35,36^) (Supplementary data S1A). We found 11 common CNAs in seminoma and non-seminoma tissues, 19 in seminomas only and 24 in non-seminomas only (Supplementary data S1A). Among them, we found the cell cycle- and CDK4/6-associated genes *CCND2* (seminomas 9.8%, non-seminomas 16.8%), *CDKN1B* (*P27*; seminomas 15.3%, non-seminomas 16.4%) and *MDM2* (seminomas 4%, non-seminomas 3.6%) commonly amplified in seminomas and non-seminomas (Supplementary data S1A). Interestingly, *CDK6* was amplified in 2.6% of seminomas, while *CCND3* (CyclinD3; 1.7%) and *CHEK1* (6.8%) were amplified or deleted in non-seminomas, respectively (Supplementary data S1A). Thus, GCT patients harbouring mutations in these cell cycle- and CDK4/6-related genes might respond differently to CDK4/6 inhibition than non-mutated patients.

**Fig. 2:**
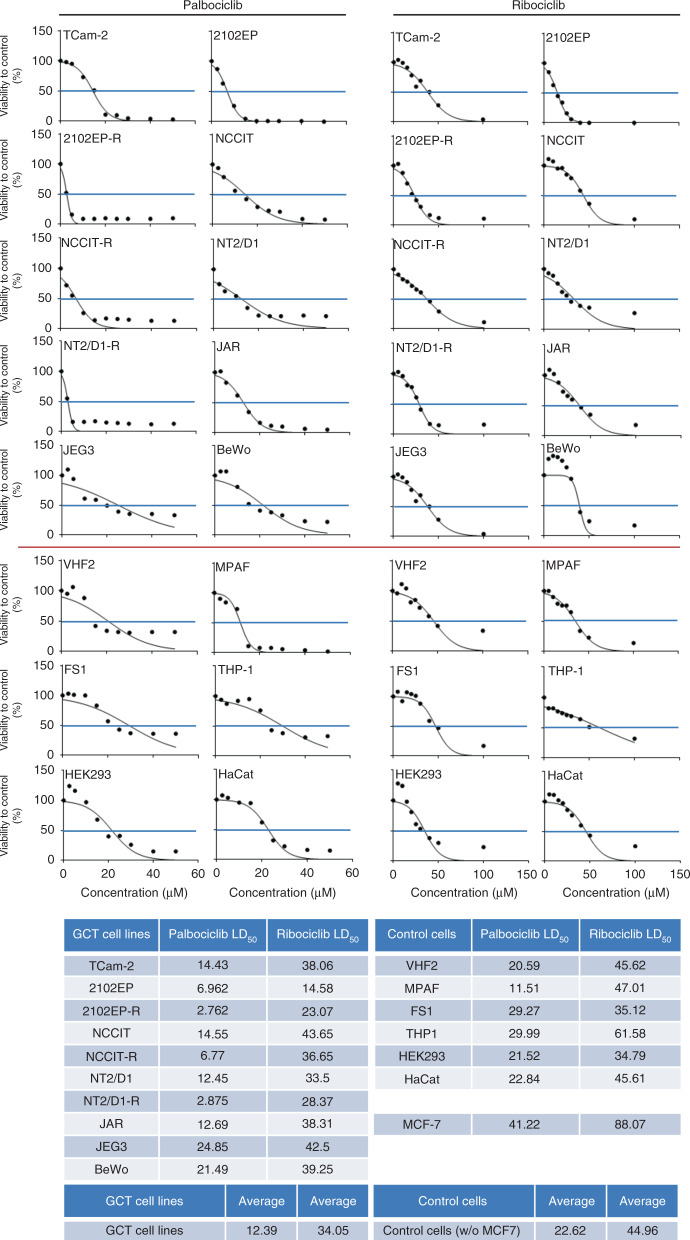
GCT cell lines are highly sensitive towards inhibition of CDK4/6. GraphPad Prism-based calculation of LD_50_ doses in GCT cell lines treated with PaRi based on XTT data (Supplementary Fig. S3). As controls, fibroblasts (VHF2, MPAF), the Sertoli cell line FS1, the monocyte cell line THP-1, the keratinocyte cell line HaCat and the kidney cell line HEK293 were included. Additionally, the breast cancer cell line MCF7 was included.

Next, we analysed the effect of PaRi on the viability of GCT cell lines by XTT assays. All GCT cell lines analysed displayed a strong reduction in viability 24–96 h after a single PaRi application (Fig. 2 and Supplementary Fig. S3). In detail, the GCT cell lines presented as more sensitive to palbociclib (LD_50_ range: 2.76–24.85 μM) than ribociclib (LD_50_ range: 14.58–43.65 μM), and among the different GCT entities, choriocarcinoma cell lines presented as least sensitive to PaRi (palbociclib average LD_50_: 19.67 μM; ribociclib average LD_50_: 40.02 μM) (Fig. 2). Interestingly, cisplatin-resistant EC subclones (−R) showed the highest sensitivity towards PaRi compared to their parental cells and all other tested cells (Fig. 2). As controls, fibroblasts (VHF2, MPAF), Sertoli cells (FS1), monocytes (THP-1), keratinocytes (HaCat) and kidney cells (HEK293) were included. On the average, the control cells (palbociclib average LD_50_: 22.62 μM; ribociclib average LD_50_: 44.96 μM) presented as less sensitive towards PaRi than the GCT cells (palbociclib average LD_50_: 12.39 μM; ribociclib average LD_50_: 34.05 μM) (Fig. 2).

**Fig. 3:**
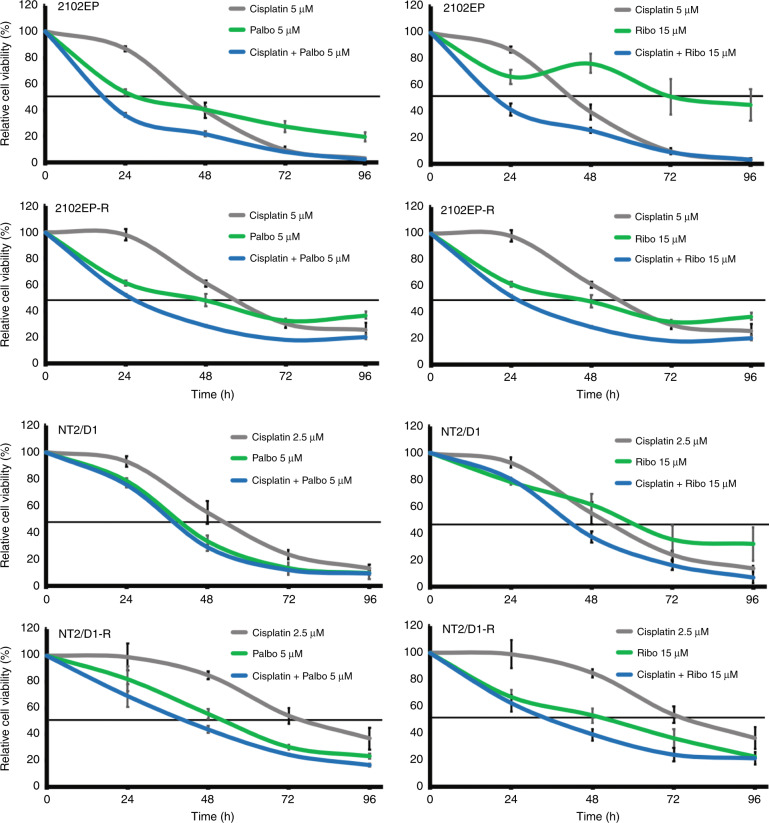
CDK4/6 inhibition enhances cisplatin efficacy in cisplatin-resistant and -sensitive GCT cells. XTT data of cisplatin-resistant and -sensitive GCT cell lines 2102EP(-R) and NT27D1(-R) treated once with palbociclib (5 μM), ribociclib (15 μM) or cisplatin (5/2.5 μM) alone and in combination. Changes in viability (compared to solvent-treated controls) were measured over 96 h.

We asked, if a combinatorial application of PaRi and cisplatin might enhance the therapeutic efficacy compared to a cisplatin-based monotherapy. Thus, we treated cisplatin-resistant and -sensitive GCT cell lines 2102EP(-R) and NT2/D1(-R) with cisplatin (5 μM/2.5 μM) alone or in combination with 5 μM palbociclib or 15 μM ribociclib (Fig. 3). Compared to the cisplatin or PaRi monotherapy, the combinatorial treatment reduced viability considerably stronger.

**Fig. 4:**
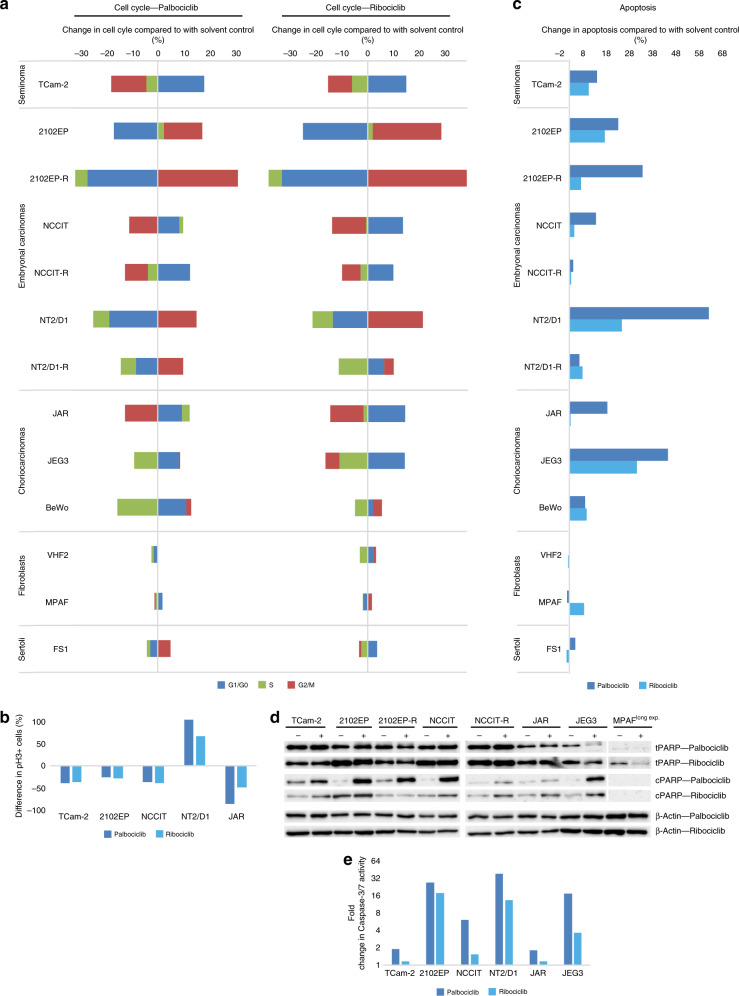
CDK4/6 inhibition affetcs the cell cycle and induces apoptosis. **a**, **b** Flow cytometry-based analysis of cell cycle phase distribution (**a**) and apoptosis rates (**b**) in GCT cell lines and controls (fibroblasts and Sertoli cells) 16 h after PaRi treatment (10 μM/25 μM). **c** Phospho-histone H3-positive (pH3+) cells were counted by flow cytometry in solvent and PaRi-treated GCT cells. Differences are given in percentage. **d** Western blot analysis of PARP (total and cleaved) protein levels in GCT cell lines and MPAF fibroblasts treated for 16 h with PaRi (10 μM/25 μM). Only a long exposure of MPAF enables detection of total PARP. β-Actin was used as housekeeper and for normalisation. **e** Changes in Caspase-3/7 activity (as fold change) in GCT cell lines after PaRi treatment compared to solvent-treated controls.

We also measured the effects of PaRi on the cell cycle and apoptosis rates by flow cytometry (Fig. 4a, b). As CDK4/6 inhibitors, PaRi should induce a semi-senescent state by blocking the cell cycle transition from G1/S phase. Interestingly, 16 h after PaRi (10 μM /25μM) treatment most GCT cell lines (TCam-2, NCCIT(-R), JAR, JEG3, BeWo) accumulated in G1/G0 phase of the cell cycle, but two EC cell lines including their cisplatin-resistant subclones accumulated in G2/M phase (2102EP(-R), NT2/D1(-R)) (Fig. 4a). The effects on the cell cycle were highly similar between palbo- and ribociclib. In control cells, the cell cycle phase distribution was affected considerably weaker (Fig. 4a). A Ki-67 immunofluorescent staining confirmed reduced proliferation rates after PaRi treatment of TCam-2, 2012EP and JAR cells (Fig. S4). To test, if the G2/M phase arrested cell lines were blocked at the end of G2 phase or enter mitosis under PaRi treatment, we counted phosphorylated histone H3-positive (pH3) cells as a marker of cells in M phase (Fig. 4b). Only NT2/D1 cells showed an increase in pH3 positive cells, while all other GCT cell lines showed a reduced number in pH3 positive cells.

PaRi considerably induced apoptosis in GCT cell lines (Fig. 4c), except for NCCIT(-R). In the control cells, nearly no apoptosis induction could be observed (Fig. 4c). We confirmed induction of apoptosis in GCT cell lines after PaRi treatment by western blot detection of cleaved PARP (Fig. 4d). In MPAF fibroblasts, no PARP cleavage could be detected, even after a prolonged exposure of the membrane (Fig. 4d). Additionally, after PaRi application we found increased activity of Caspase-3/7 in GCT cell lines (Fig. 4e). The activity levels resembled the levels of apoptosis induction found in the Annexin V/PI analysis (Fig. 4c vs. e).

Accumulation of GCT cells in either in the G1/G0 or G2/M phase of the cell cycle and the induction of apoptosis pointed at an alternative mode of action of PaRi apart from the canonical CDK4–RB1 axis, prompting us to decipher the molecular mode of action of PaRi in GCT cells in more detail. We performed RNA-sequencing (RNA-seq) analyses of GCT cells (TCam-2, 2102EP, NCCIT, JAR, JEG3 and fibroblasts (MPAF)) treated for 16 h with 10 μM palbociclib to measure transcriptome-wide changes in gene expression. High quality (RNA quality number = 10) of all sequenced RNAs was verified by Agilent Fragment Analyser (Supplementary Fig. S5A). A principal component analysis demonstrated that palbociclib led to the strongest differences in the transcriptome in the choriocarcinoma cell lines JEG3 and JAR and the EC cell lines 2102EP and NCCIT followed by TCam-2, while MPAF fibroblasts displayed the weakest effects (Supplementary Fig. S5B).

**Fig. 5:**
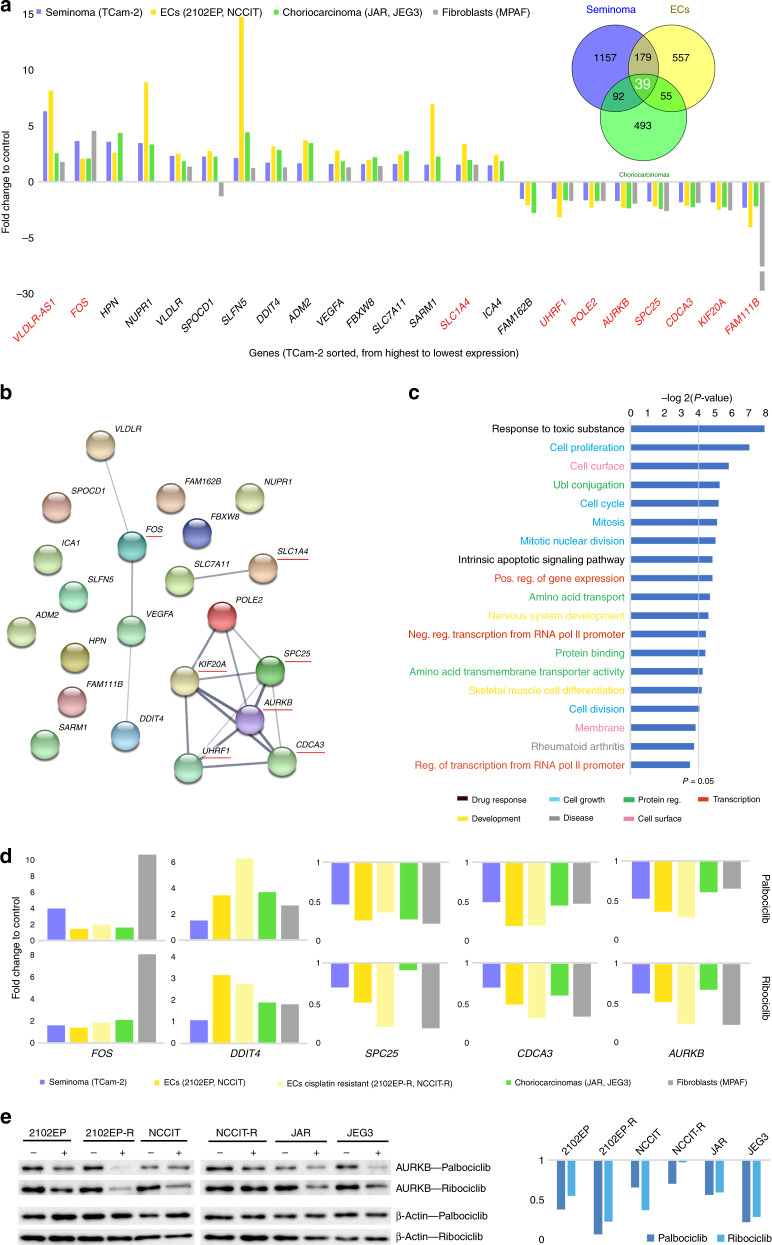
Deciphering the molecular mechanisms of PaRi. **a** RNA-seq identified commonly deregulated transcripts in GCT cell lines 16 h after 10 μM palbociclib treatment. In MPAF control cells, 10 of the 23 transcripts were also deregulated (≥fold change 1.5) after palbociclib application (red labelled genes). **b** STRING-based interaction prediction of the commonly deregulated genes. Genes also deregulated in fibroblasts are labelled in red. **c** DAVID-based GO analysis of the commonly deregulated genes. **d** Validation of RNA-seq data by qRT-PCR in GCT cell lines and MPAF fibroblast treated with PaRi (10 μM/25 μM) for 16 h. *GAPDH* was used as housekeeper. Data are given as fold changes (PaRi treated vs. solvent control). Standard deviation is given above bars. **e** Validation of AURKB downregulation on protein level by western blotting. GCT cells were treated with PaRi (10 μM/25 μM) for 16 h. β-ACTIN was used as housekeeper. Densitometric evaluation of western blot data is given on the right side.

We identified all transcripts deregulated (*p* values ≤ 0.05, fold change ≥ 1.5) in GCT cell lines after palbociclib treatment compared to solvent-treated cells (Supplementary data S1B). Among them, 39 transcripts were deregulated in all GCT cell lines tested (Fig. 5a, Venn diagram; Supplementary data S1C).^31^ We excluded all transcripts that could not be matched to an official gene symbol and were differentially expressed between the GCT cell lines, to end up with 23 commonly deregulated genes, of which 15 were upregulated and 8 downregulated (Fig. 5a). Ten of these genes were also deregulated in MPAF fibroblasts (Fig. 5a, red labelled genes, fold change ≥1.5). We used the STRING algorithm to identify putative interactions (Fig. 5b).^32^ Basically, three interactive networks were predicted: (1) consisting of *VLDLR*, *FOS*, *VEGFA* and *DDIT4*, (2) consisting of *SLC7A11* and *SLC1A4* and (3) consisting of *POLE2*, *KIF20A*, *AURKB*, *UHRF1* and *CDCA3*. Interestingly, genes also deregulated in MPAF fibroblasts (underlined in red) were mainly found in network (3). Based on a DAVID gene ontology (GO) analysis, the commonly deregulated genes in GCT cells could be associated with (ranked by frequency) cellular growth processes (genes in network (3)), such as mitosis, cell cycle and proliferation (light blue), protein regulatory processes (light green), regulation of transcription (red), drug response (black), developmental processes (yellow), cell surface (pink) and disease (grey) (Fig. 5c and Supplementary data S1D).^33,34^ Thus, downregulation of the network (3) genes (*SPC25*, *KIF20A*, *AURKB*, *UHRF1* and *CDCA3*) is a common palbociclib response in GCT cell lines and fibroblasts affecting the cell cycle. We confirmed RNA-seq data by qRT-PCR analysis of *FOS*, *DDIT4*, *VEGFA*, *UHFR1* and *AURKB* in palbociclib-treated GCT cell lines and fibroblasts (Fig. 5d). Additionally, we confirmed downregulation of AURKB in palbociclib-treated, cisplatin-resistant and -sensitive GCT cells by western blotting (Fig. 5e). Importantly, we demonstrated that these genes/proteins become deregulated in a similar way after ribociclib treatment of GCT cells and MPAF, arguing further for a common effect of CDK4/6 inhibition (Fig. 5d, e).

Next, we analysed the individual effects of palbociclib on each GCT entity (seminoma, ECs, choriocarcinomas) and MPAF fibroblasts by a DAVID GO analysis (Supplementary Figs. S6 and S7A). Again, it became obvious that mostly genes associated with the cell cycle were deregulated (Supplementary Figs. S6 and S7A; labelled in light blue), which is in line with the expected molecular effect caused by the palbociclib-mediated CDK4/6 inhibition (pseudo-senescent cell state; G1/S-phase arrest). To narrow down the cell type-/tumour entity-specific effects in more detail, we performed a STRING analysis of all genes related to the GO term ‘cell cycle’ and ended up with networks, presumably representing the key factors of each entity that regulate/influence the cell cycle in response to CDK4/6 inhibition (Supplementary Fig. S6, Supplementary data S1E).

Independent of our set thresholds, we also screened for expression of cell cycle-related factors like cyclins, CDKs, CDK inhibitors and necessary co-factors (Supplementary data S1F). Here, we observed a common trend in the upregulation of *CCND1/2/3*, *CDK6/7* and *E2F5*, while *CCNA2*, *CCNB1/2*, *CDK1*, *CDKN2D/3*, *CDC2/3/4/7/20/25A/25B/25C* and *E2F1/2* were downregulated (Supplementary Fig. S7B). Mostly, in MPAF fibroblasts the same trends in deregulation were observed (except for *CCND2/D3* and *CDK6*) (Supplementary Fig. S7B). Thus, PaRi induce quite similar deregulations in canonical cell cycle regulators in GCT and fibroblast cells. We confirmed downregulation of CCNA2 and CCNB1 in PaRi-treated, cisplatin-resistant and -sensitive GCT cell lines by western blotting (Supplementary Fig. S7C). In contrast to upregulation of RNA level, CCND1/D2 were not altered on protein levels in ribociclib-treated GCT cell lines (Supplementary Fig. S7C).

Of note, PaRi treatment led to an increased CDK4 protein level in most GCT cell lines, but not in MPAF fibroblasts, pointing at a putative PaRi counteracting mechanism in GCT cell lines (Supplementary Fig. S7D).

From the cell cycle analysis, we observed that 2102EP(-R) and NT2/D1(-R) accumulated in the G2/M phase instead of the G1/G0 phase of the cell cycle (Fig. 4a). To identify factors that might dictate this decision, we analysed all genes related to the GO term ‘cell cycle’ and are exclusively expressed in 2102EP cells by the STRING algorithm (Supplementary Fig. S8A, Supplementary data S1G). Within this group, we found genes involved in the G2/M transition in the cell cycle (Supplementary Fig. S8A). Two genes (*NAE1*, *HMGA2*) were linked to all mentioned GO categories, six genes (*CDC25C*, *CDC7*, *CNTRL*, *HAUS1*, *HAUS2*, *HAUS6*) were linked to four out of five detected categories, two genes (*CDK3, CHEK1*) were linked to three out of five categories and five genes (*TERF1*, *BORA*, *PPM1D*, *MASTL, PLK3*) were related to two out of five categories (Supplementary Fig. S8A). Of these 15 genes, 13 were downregulated and two upregulated (*CDK3*, *PLK3*) in expression (Supplementary Fig. S8B). Interestingly, CDK3 is able to promote G1/S checkpoint transition in an RB1-independent manner.^38^

Taken together, some EC cells are able to bypass the PaRi-induced G1/S arrest and reach the G2/M phase of the cell cycle, but downregulation of a plethora of the G2/M phase-regulating genes impedes completion of cell cycle and leads to induction of apoptosis.

## Discussion

In this study, we demonstrated the utility of CDK4/6 inhibitors palbociclib and ribociclib in treating cisplatin-resistant and -sensitive GCTs. GCTs presented as more sensitive towards PaRi than analysed control cells (including MCF7 breast cancer cells), opening a putative therapeutic window for patients suffering from GCT. Although, it has to be mentioned that results gathered in this study by comparing GCT cell lines to one breast cancer cell lines is not enough to draw reliable conclusions on the differences between GCTs and breast cancers regarding PaRi treatment. In the future, comparing large tumour cohorts of each entity would be necessary.

Among the different GCT entities, choriocarcinoma cells presented as least sensitive and EC cells as most sensitive. Interestingly, Schmidt et al.^18^ demonstrated that among seminomas and non-seminomas, ECs show highest levels of *CDK4* expression, thus in ECs sensitivity to CDK4/6 inhibition correlates to high levels of *CDK4* expression.^18^ Additionally, in our study cisplatin-resistant EC subclones showed a further increased sensitivity compared to parental cells. This suggests that mechanisms causing cisplatin resistance might in turn cause sensitisation towards inhibition of cell cycle regulators, that is, CDK4 or application of PaRi is able to revert the cisplatin-resistant phenotype. In line, combination of cisplatin with PaRi might be a novel therapeutic option for treatment of GCT patients, since combining both drugs considerably increased therapeutic efficacy. It remains elusive if combining PaRi with cisplatin leads to additive or synergistic effects.

So far, only one early clinical study (phase 2, 29 patients) tested the potential of the CDK4/6 inhibitor palbociclib in treating ﻿incurable, refractory, RB1-expressing GCTs.^39^ In this study, especially mature teratomas (including cases with malignant transformation) had a favourable 24-week progression-free survival rate.^39^ In a follow-up study, ﻿Narayan et al.^40^ performed a retrospective analysis with long-term follow-up data of the patient cohort with unresectable mature teratoma treated with palbociclib and demonstrated that in 12 patients ﻿palbociclib might lead to a clinically meaningful delay in disease-related clinical events. Further, a randomised, blinded and placebo-controlled clinical study (NCT02300987) analysed the suitability of ribociclib to treat relapsed, refractory, incurable teratoma with recent progression. In this study, only 10 participants were recruited and thus numbers are quite limited (results pending). Nevertheless, CDK4/6 inhibition seems to be promising in treating especially mature teratomas, including teratomas with malignant transformation. The beneficial effect of palbociclib on the progression-free survival of non-teratoma GCT patients seems to be considerably weaker compared to patients with teratoma.^39^ Nevertheless, in the study of Vaughn et al.^39^, only a limited number of patients as well as only RB1-expressing GCT were included and only palbociclib was tested. Based on the result of our study, in GCTs PaRi do not only act via the canonical CDK4–RB1 axis and provoke effects beyond a cell cycle block, that is, apoptosis. Thus, it would be interesting and worth to (a) screen a larger cohort of type I and type II GCT patients with (b) no restrictions to RB1 status and to (c) include ribociclib and abemaciclib (Lilly) as alternatives to palbociclib. Additionally, we found that PaRi treatment led to an upregulation of CDK4 in GCT cell lines, which we postulated might counteract the effects of PaRi and might cause resistance. It would be interesting to screen the patients treated by Vaughn et al.^39^ for upregulation of CDK4 under therapy. If upregulation of CDK4 would be restricted to non-teratomatous GCT patients, the reduced activity of palbociclib in these patients could be explained.

In type II adult GCT cells *CDK4* was highly expressed, while *CDK6* expression was very low, confirming results of Schmidt et al.,^18^ who also detected high CDK4 in 41% of GCTs (most strongly in ECs) and reduced CDK6 expression in 64% of seminomas and 23% of non-seminomas. Thus, in adult type II GCTs CDK4 seems to be the primary target of PaRi. In contrast, in all type I paediatric GCTs analysed, *CDK6* was expressed considerably higher than *CDK4*. In conclusion, although differing in expression of PaRi target molecules, PaRi might be a therapeutic option for both type I and type II GCTs.

**Fig. 6:**
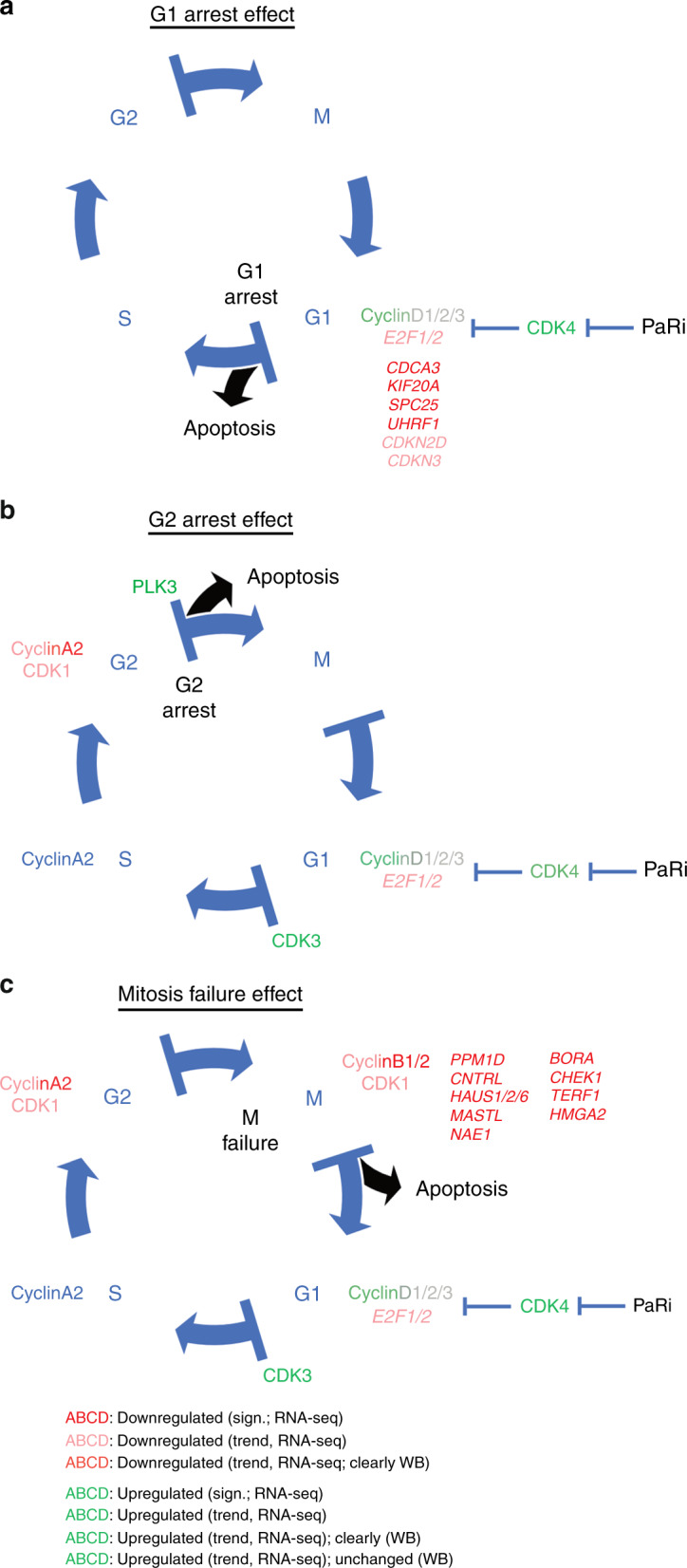
Model summarising the results of this study. In GCTs, PaRi affects the cell cycle in three ways: **a** by inducing G1/G0 arrest via the CDK4–pRB1 axis, **b** by bypassing the G1/S checkpoint by upregulation of CDK3 and inducing an arrest at the end of the G2 phase or **c** by downregulating mitosis regulators leading to termination of mitosis. All described effects are not tolerated by GCT cells, eventually resulting in apoptosis.

In type II GCT cell lines, PaRi induced a pseudo-senescent state, mostly leading to accumulation of cells in the G1/G0 phase of the cell cycle (Fig. 6a). Nevertheless, especially EC cells seem capable of escaping the G1/G0 arrest and continuing the cell cycle (Fig. 6b, c). Our results suggested that EC cell lines either accumulate at the end of the G2 phase or are able to enter mitosis, but without completing this process (Fig. 6b, c).

Furthermore, PaRi induced apoptosis in cisplatin-resistant and parental GCT cell lines, but not in the tested control cells. Induction of apoptosis after PaRi has also been described in bladder cancer cells and T cell acute lymphoblastic leukaemia, while in pancreatic ductal adenocarcinoma cell lines, CDK4/6 inhibition alone did not induce apoptosis.^41–43^ Thus, induction of apoptosis in response to CDK4/6 inhibition seems to be tumour type dependent, but is in general of benefit in terms of a therapeutic application.

On a molecular level, PaRi considerably affected cell cycle-related genes in GCT cells, but interestingly factors that mediate this response were different between the GCT entities. Although regulators of different cell cycle phases become deregulated upon PaRi application, GCTs mostly accumulate already in the G1/G0 phase. This G1/0 phase accumulation seemed to be the result of downregulation of early cell cycle phase regulators *E2F/1*, *CDCA3*, *KIF20A*, *SPC25*, *UHRF1*, *CDKN2D* and *CDKN3* (Fig. 6a).

Interestingly, CDK3 is able to promote the G1/S-phase transition in a RB1-independent manner^38^ (Fig. 6b). Thus, in EC cells, upregulation of *CDK3* might enable progression beyond the G1/S-phase checkpoint, independent of the palbociclib-inhibited CDK4–RB1 axis (Fig. 6b). Then, EC cells either arrest at the end of the G2 phase or enter mitosis without completing this process (Fig. 6b, c). PLK3 regulates the M phase of the cell cycle by interacting with CDC25C (becomes inactivated by PLK3) and CHEK1. After palbociclib treatment, *PLK3* was upregulated in 2102EP cells, while *CDC25C* and *CHEK1* were downregulated (Supplementary Fig. S8A, B). Thus, upregulation of *PLK3* might lead to downregulation of *CDC25C* and *CHEK1*. Additionally, *CHEK1* is deleted in 6.8% of non-seminomas, suggesting that deletion of *CHEK1* might be of benefit for efficacy of a CDK4/6 inhibitor treatment in non-seminomas (Supplementary data S1A). Furthermore, downregulation of the human augmin complex genes *HAUS1*/*2*/*6*, which play an important role in mitotic spindle and centrosome integrity, was observed after PaRi application (Supplementary Fig. S8A, B). These effects combined might prevent the EC cells from completing mitosis (Fig. 6b, c). Independent of the cell cycle state, the cells arrested in apoptosis was induced in GCT cell lines after PaRi application, suggesting that even cells that enter mitosis do not complete this process. We conclude that GCT cells are not able to tolerate the pseudo-senescent state (either at G1/S checkpoint or when accumulating at the end of G2 phase) or a misregulated mitosis and undergo apoptosis soon after (Fig. 6a–c). Thus, we hypothesise that induction of apoptosis is not a directly provoked effect by PaRi, but a consequence of the cells’ inability to tolerate the prolonged alterations in the cell cycle.

We observed that CDK4 is upregulated on protein level after PaRi treatment. We propose that GCT cells try to counteract inhibition of CDK4 by upregulating its expression, leading to the production of new (and more) CDK4. Thus, GCT cells might become resistant to PaRi treatment over time, but we found no upregulation of the CDK4 downstream target *CCND1*/*CCND2*. Upregulation of CCND in response to CDK4/6 inhibition has been shown in several publications and was linked to acquired resistance by bypassing cytostasis via a CyclinD1-CDK2-mediated S-phase entry.^12,44,45^ GCT cell lines seem to bypass this mechanism as GCT cell lines predominately accumulated in the G1/G0 phase of the cell cycle after PaRi treatment and neither upregulated CyclinD1/2 on protein level nor significantly upregulated *CDK2* on RNA level (Supplementary Fig. S7C, Supplementary data S1B).

In our analysis of CNAs, we found that *CCND2* is commonly amplified in seminomas and non-seminomas (Supplementary data S1A). Maybe, those patients are more prone to become resistant to CDK4/6 inhibition than non-*CCND1*-amplified patients. Advances in PROteolysis TArgeting Chimeras (PROTACs) suppressing CDK4/6 were recently introduced in breast cancer and glioblastoma cell lines, where CDK4 and CDK6 protein expressions were reduced considerably.^46^ Thus, a PROTAC targeting CDK4/6 could be used as an alternative to PaRi, if resistance to these drugs might be an issue.

## Conclusions

In conclusion, palbociclib and ribociclib present a new putative therapeutic option for cisplatin-resistant and -sensitive paediatric and adult GCTs. Nevertheless, it has to be shown in the future whether mechanisms and drug kinetics/dynamics found and measured in this study on artificial cell lines can be mirrored to the in vivo situation. Therefore, large type I and II GCT cohorts have to be analysed in detail. Additionally, further experiments will have to reveal whether GCTs are able to acquire resistance towards PaRi and if a combinatorial treatment of cisplatin and PaRi can be of benefit for the patients. Up to now, it remains elusive if CDK4/6 inhibition might revert a cisplatin-resistant phenotype or if mechanisms causing cisplatin resistance in parallel sensitise GCT cells towards drugs that interfere with the cell cycle.

## Supplementary information

Supplemental Figures, combined word-file

Data S1

## Data Availability

All data published in this study is available upon request to the corresponding author. Illumina gene expression microarray data are available via GEO (ncbi.nlm.nih.gov/geo/) (GSE71239, GSE71269, GSE79065, GSE60698). The Illumina 450k DNA methylation array data is also available via GEO (GSE76709). The Cancer Genome Atlas’ (TCGA) datasets can be accessed via the UCSC Xena browser (https://xena.ucsc.edu) and cBioPortal (https://www.cbioportal.org).
