# Supplementary material for: CDK4/6 inhibition presents as a therapeutic option for paediatric and adult germ cell tumours and induces cell cycle arrest and apoptosis via canonical and non-canonical mechanisms
Source: Br J Cancer. 2020 May 18;123(3):378–91. doi: 10.1038/s41416-020-0891-x (PMC7403155; doi:10.1038/s41416-020-0891-x)
Supplement: Supplementary file 1 — Supplemental Figures, combined word-file [file 41416_2020_891_MOESM1_ESM.pdf]

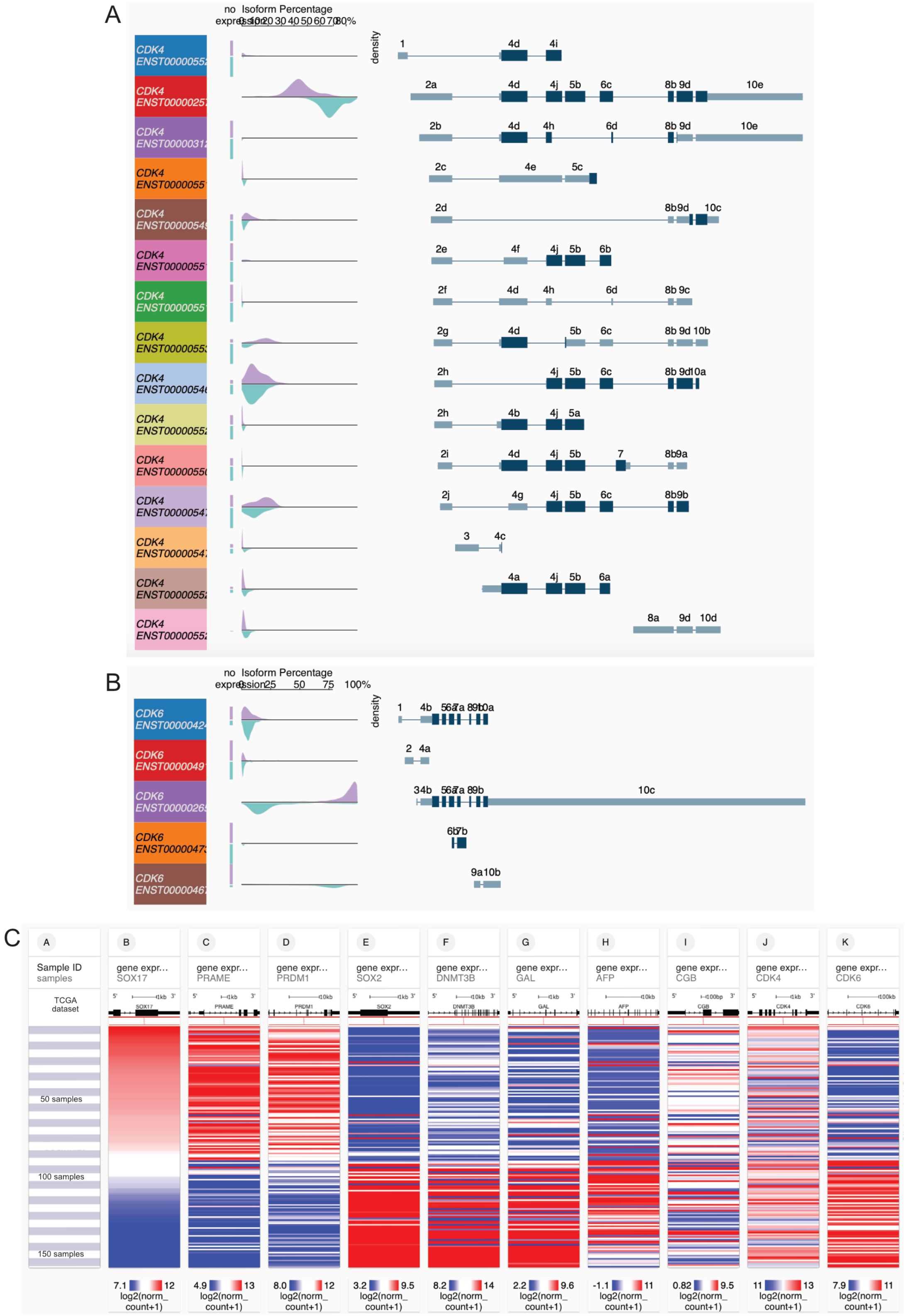

Figure S1:
A, B) UCSC Xena browser-mediated analysis of *CDK4* (A) and *CDK6* (B) isoform expression in GCT tissues (purple) and normal testis tissue (green). C) TCGA datasets were stratified in a seminoma (*SOX17*, *PRAME*, *PRDM1* positive) and EC (*SOX2*, *DNMT3B*, *GAL* positive) signature. *AFP* and *beta-hCG* positivity indicates yolk-sac tumor and choriocarcinoma / trophoblast components, respectively. *CDK4* and *CDK6* expression was analyzed in context of this study.

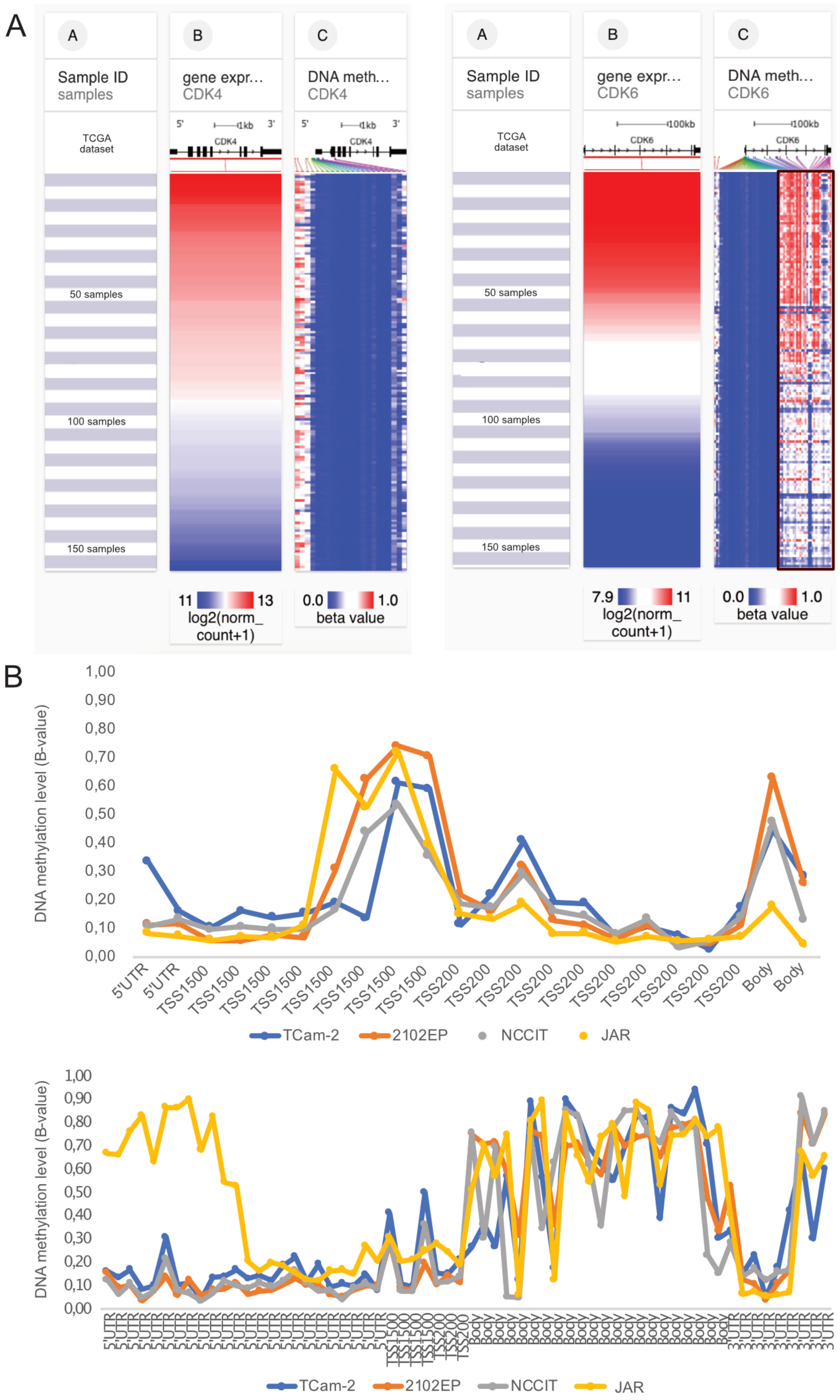

Figure S2:

A) UCSC Xena browser-mediated correlation analysis of *CDK4* and *CDK6* expression to DNA methylation levels (Illumina 450k arrays data) in GCT tissues. B) DNA methylation profile of *CDK4* / *6* in indicated GCT cell lines (data re-analyzed from (31).

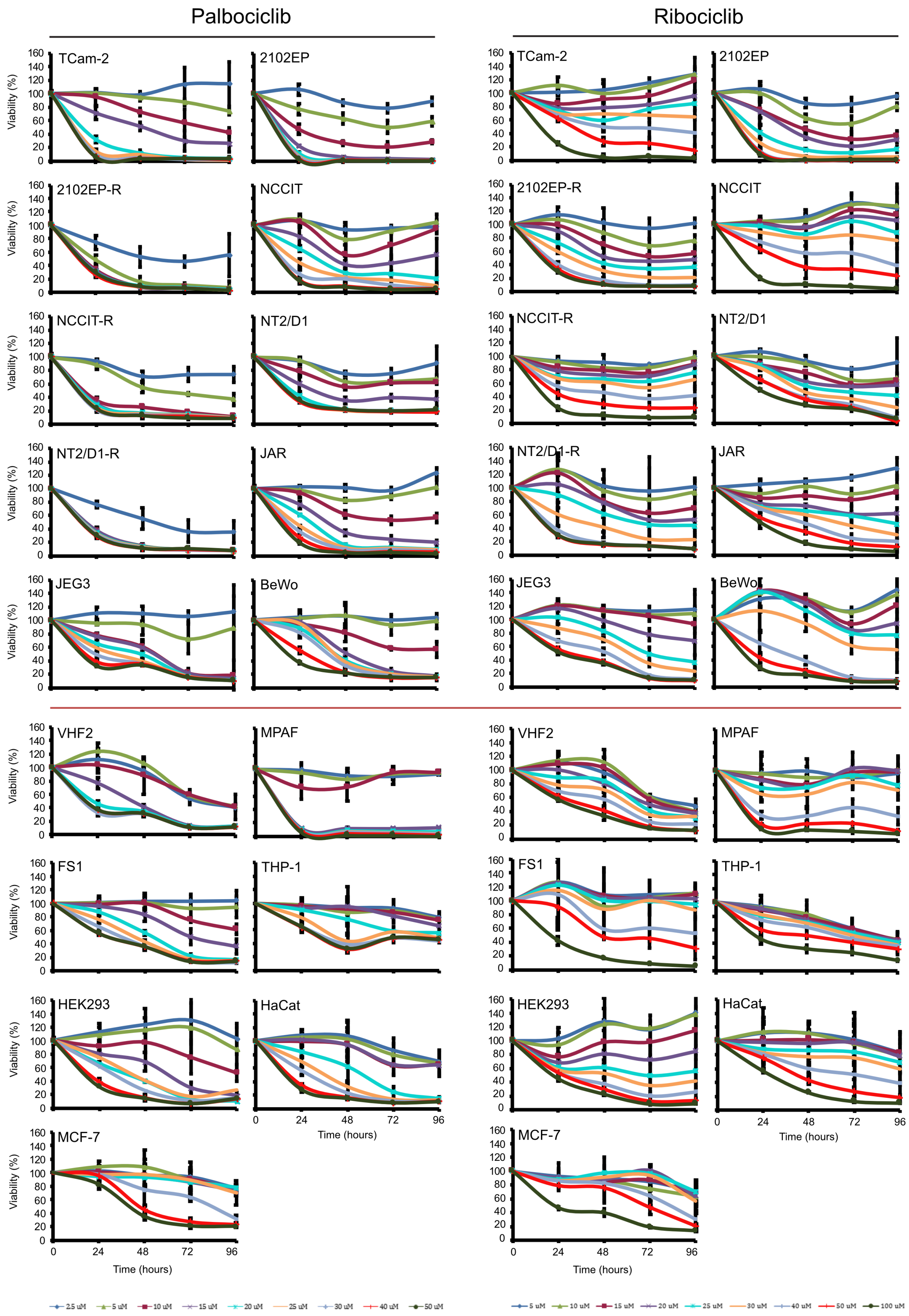

Figure S3:
XTT data of GCT and control cells treated once with palbociclib (2.5 - 50 μM) and ribociclib (5 - 100 μM). Changes in viability (compared to solvent treated controls) were measured over 96 h. Standard deviation is given above each point.

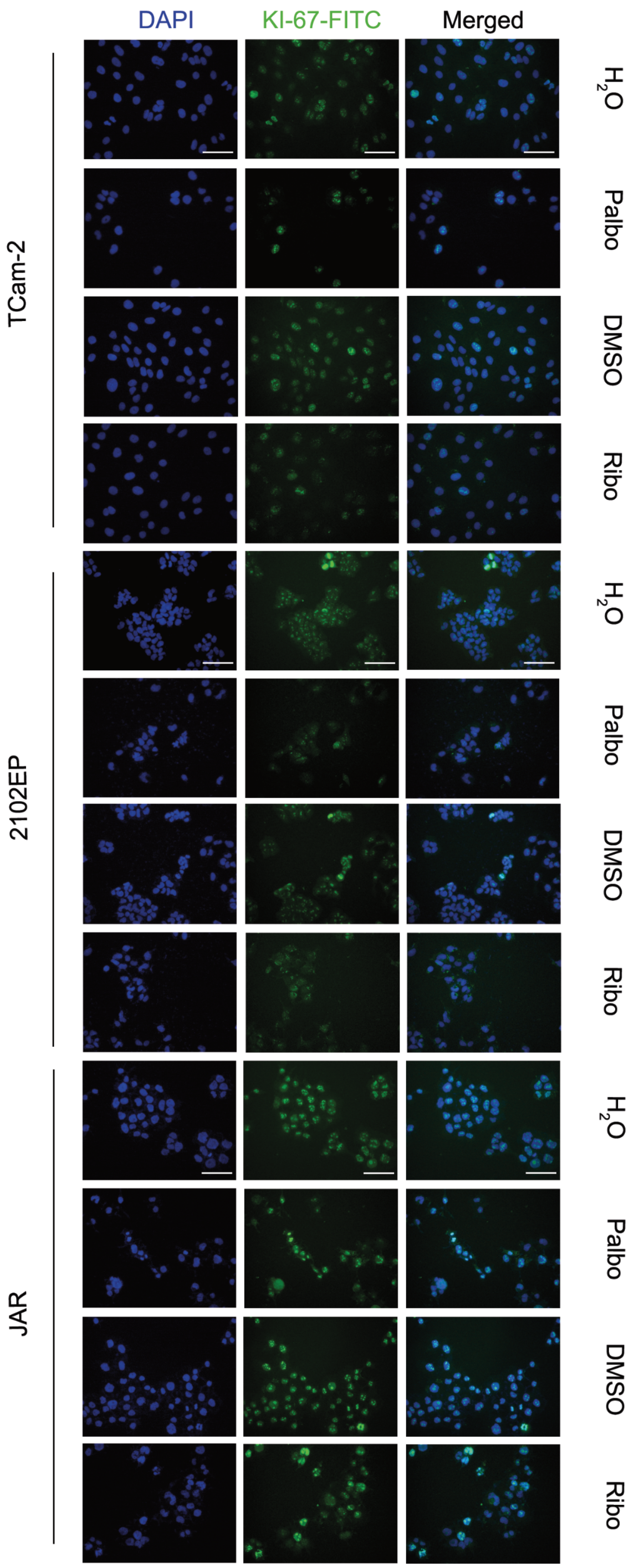

Figure S4:
Immunofluorescent staining of Ki67 in GCT cell lines TCam-2, 2102EP and JAR treated with PaRi (10 µM / 25 µM for 16 h). Nuclei were counterstained by DAPI. Scale bar: 100 µm.

A

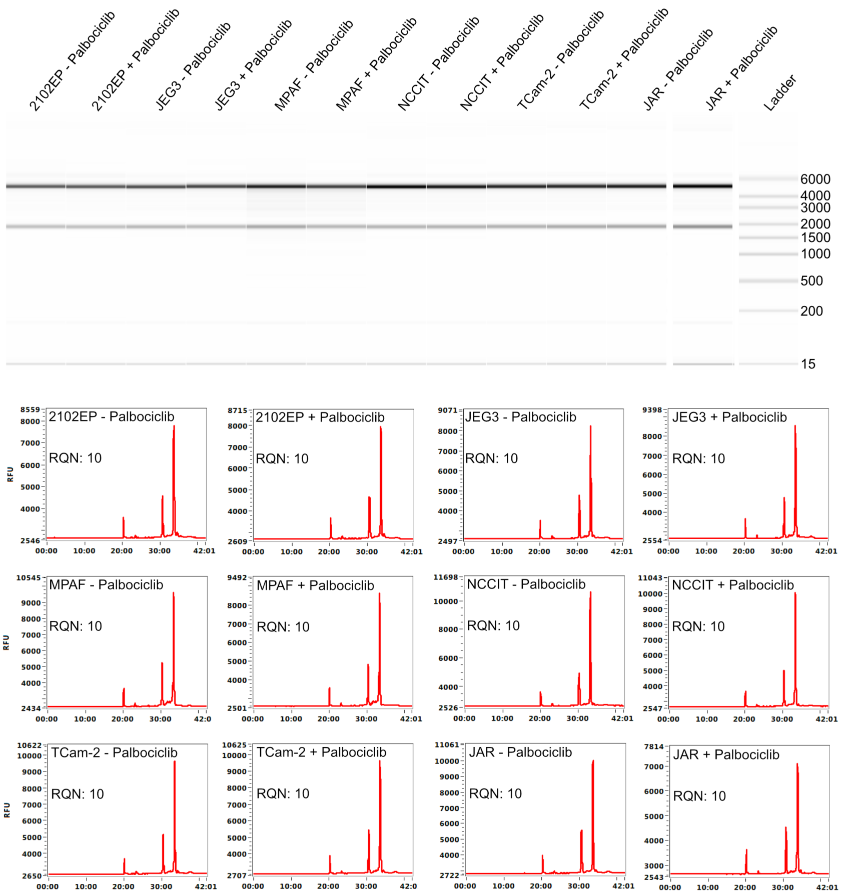

B

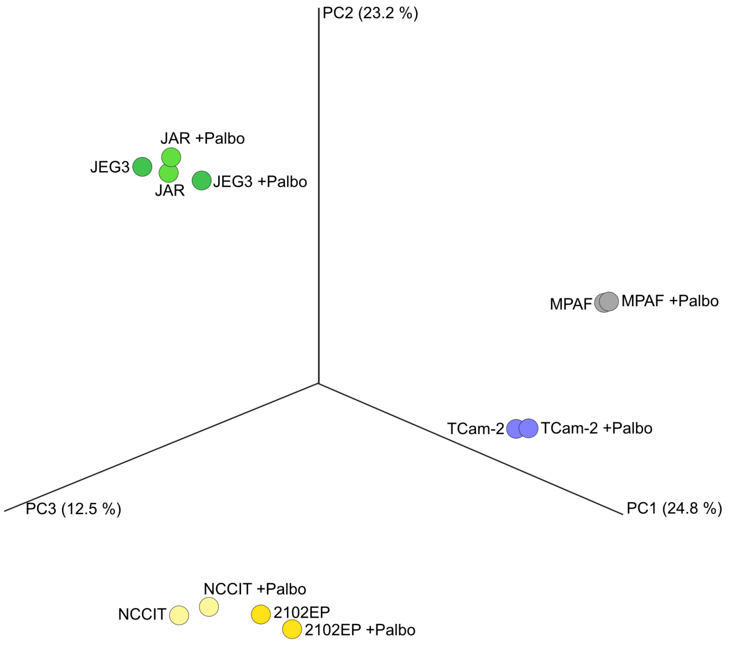

Figure S5:
A) Results of the Agilent Fragment Analyzer quality control step for each isolated RNA used for RNAseq. Only RNA with a RNA Quality Number (QRN) of 10 was used for subsequent analyses. B) PCA analysis of RNAseq datasets of GCT cells and fibroblasts treated with 10 μM palbociclib for 16 h. Palbociclib induced only moderate changes in the transcriptome of analyzed cell lines.

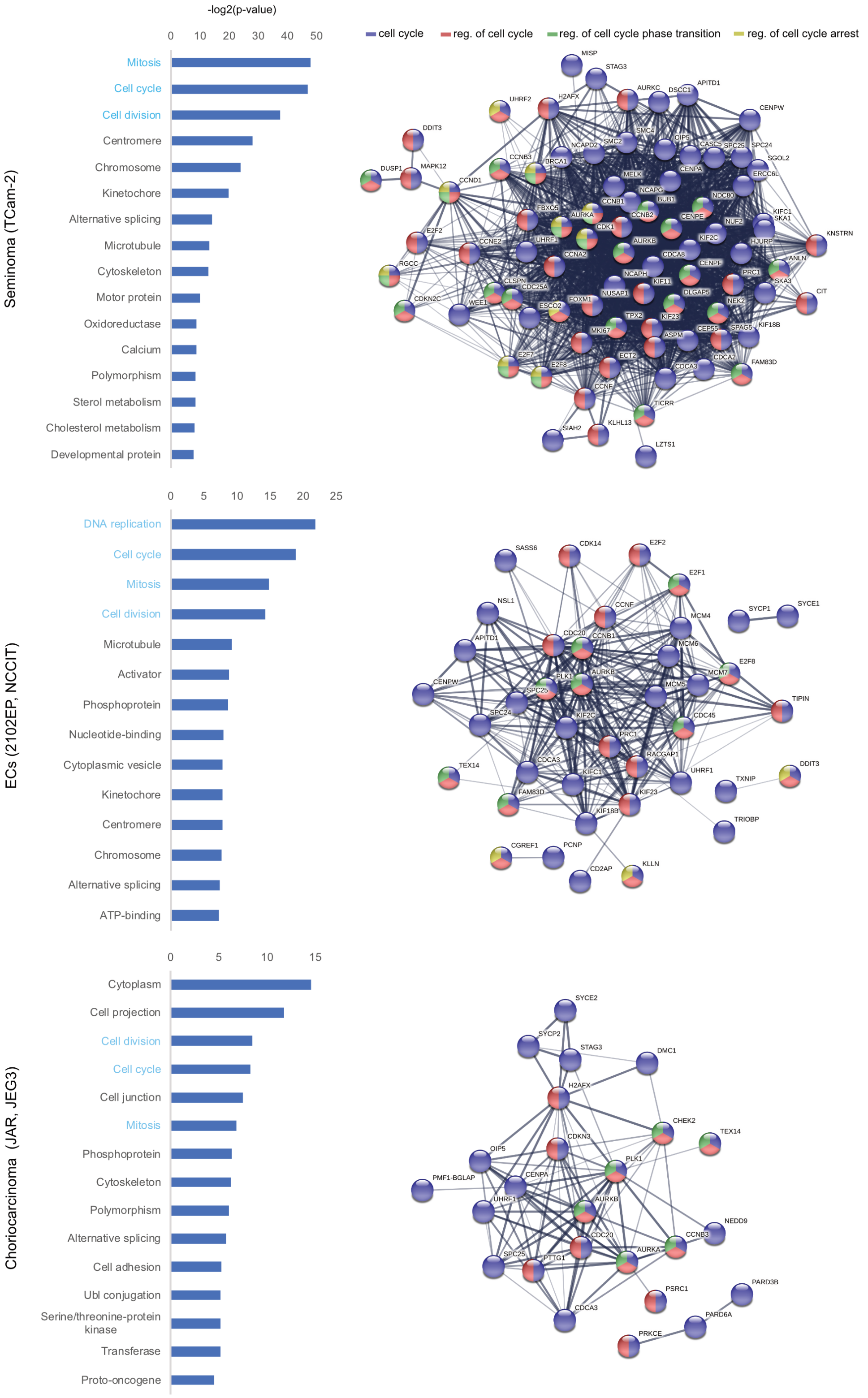

Figure S6:
DAVID-based GO analyses of genes significantly deregulated in seminomas (TCam-2), ECs (2102EP, NCCIT) or choriocarcinomas (JAR, JEG3) are given on the left side. In each GCT entity, deregulated genes could be attributed to GO categories ‘Mitosis’, ‘Cell cycle’, ‘Cell proliferation’. On the right side, STRING interaction analyses of all genes related to the GO category ‘Cell cycle’ from the DAVID analyses (blue). Contribution to additional GO categories associated with regulation of the cell cycle are highlighted in different colors (red, green, yellow).

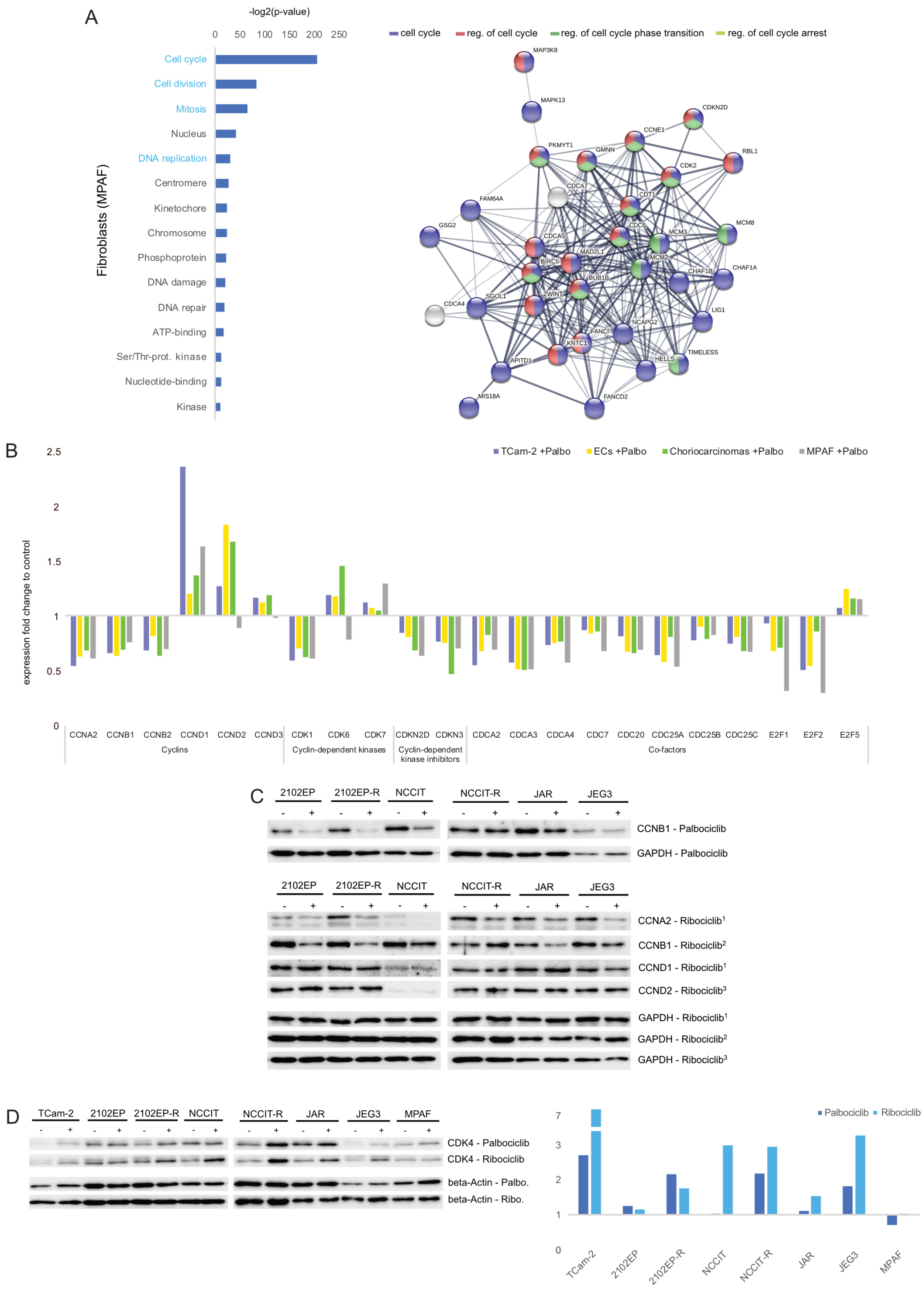

Figure S7:
A) DAVID-based GO analyses of genes significantly deregulated in MPAF fibroblasts. B) Expression fold changes (compared to solvent control) of indicated cell cycle-related genes commonly deregulated in GCT cells treated for 16 h with 10 µM palbociclib. Fibroblasts were included as non-cancerous cells. No threshold regarding *p*-value or minimum fold change was set. For extended data regarding cell cycle-associated genes, see Data S1 E. C) Western blot analysis of indicated cell cycle regulators in GCT cell lines treated with PaRi (10 µM / 25 µM for 16 h). GAPDH was used as housekeeper (numbers indicate corresponding samples). D) Western blot analysis of CDK4 protein levels in GCT cell lines and MPAF fibroblasts treated for 16h with PaRi (10 µM / 25 µM). Beta-ACTIN was used as housekeeper. A densitometric evaluation of CDK4 protein levels (PaRi treated vs. solvent controls) is given on the right side.

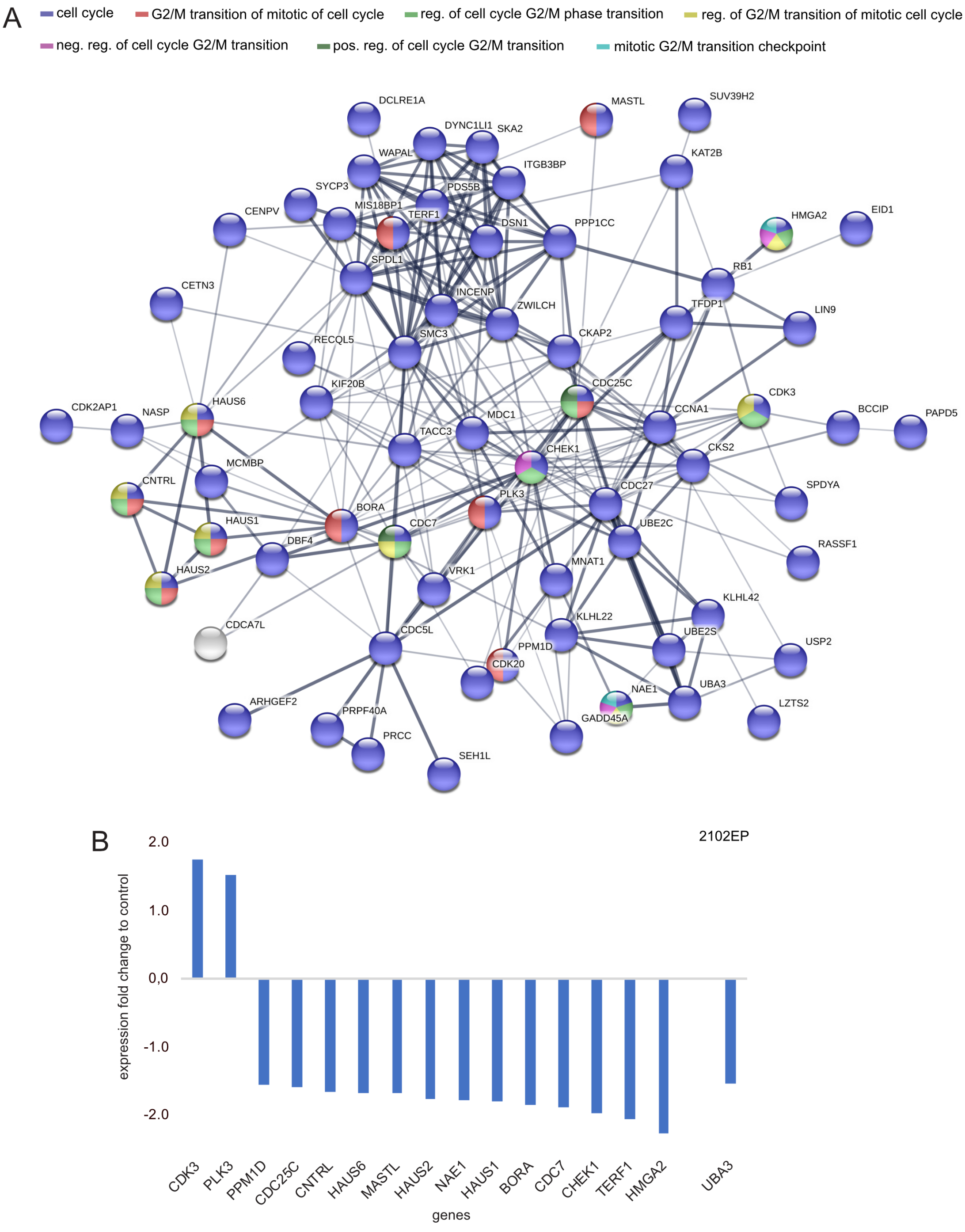

Figure S8:
A) STRING-based interaction analysis of genes deregulated in 2102EP cells only after palbociclib treatment and associated with the GO category ‘cell cycle’ (blue). These factors were screened for involvement in different G2/M phase associated GO-processes (various colors). B) Waterfall diagram of expression of these G2/M phase associated factors (related to at least two GO categories) in 2102EP cells after 10 μM palbociclib treatment.

Data S1:
CNA analysis of GCT tissues and evaluation of RNAseq data of palbociclib treated GCT cell lines
